# Public–pastoralist perception gaps in valuing pastoralism: Implications for its sustainability

**DOI:** 10.1007/s13280-026-02401-2

**Published:** 2026-04-29

**Authors:** F. Javier Pérez-Barbería

**Affiliations:** Biodiversity Research Institute (Spanish Research Council - University of Oviedo - Principality of Asturias), Universidad de Oviedo-Campus de Mieres, Edificio de Investigación, 33600 Mieres, Asturias Spain

**Keywords:** Livestock ecosystem services, Pastoralism, Public perceptions, Rural–urban divide, Social acceptance, Sustainability

## Abstract

Pastoralism remains vital to Mediterranean landscapes and cultural heritage; yet, pastoralists increasingly doubt how far society accepts and values their activity. Using a nationwide survey of the Spanish public, I examined social attitudes toward pastoralism and compared them with recent surveys of pastoralists in the same region. I found that rural background and older age are associated with more favorable views of pastoral and other extensive livestock systems, while intensive systems are viewed least favorably. The public reports declining social appreciation of pastoralism over time, but relatively infrequent conflict and low concern about externalities. In contrast, pastoralists perceive strong urban intolerance and a widespread loss of social esteem. This comparison reveals that citizens are more accepting of pastoralism than pastoralists believe. I highlight this crucial perception gap and identify communication, policy, and governance measures that support the long-term sustainability of the largest pastoralist system of Europe.

**Graphical abstract:**

**Figure:**
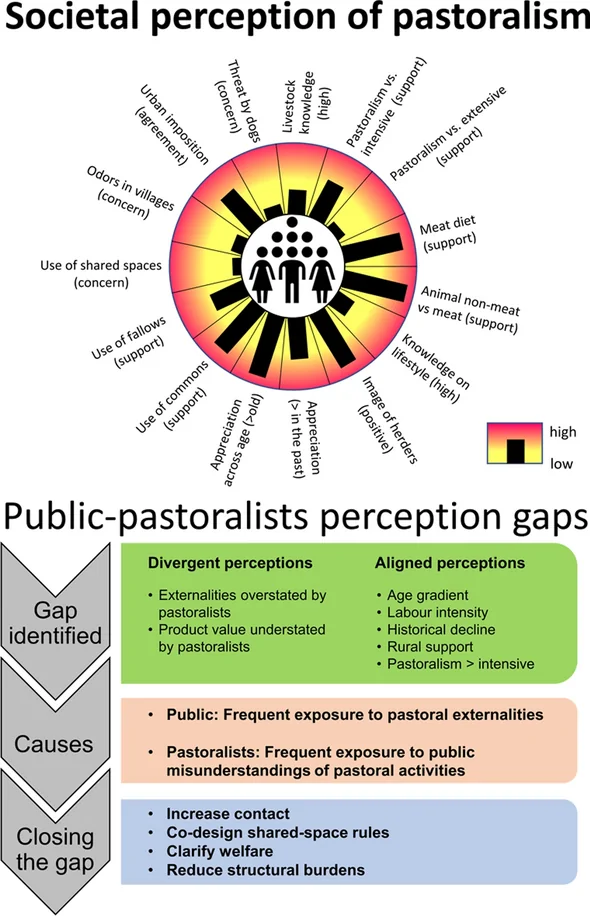
Synthesis of questionnaire responses (top) and of the main perception gaps between the public and pastoralists, along with their potential causes and proposed solutions (bottom).

**Supplementary Information:**

The online version contains supplementary material available at https://doi.org/10.1007/s13280-026-02401-2.

## Introduction

**Fig. 1:**
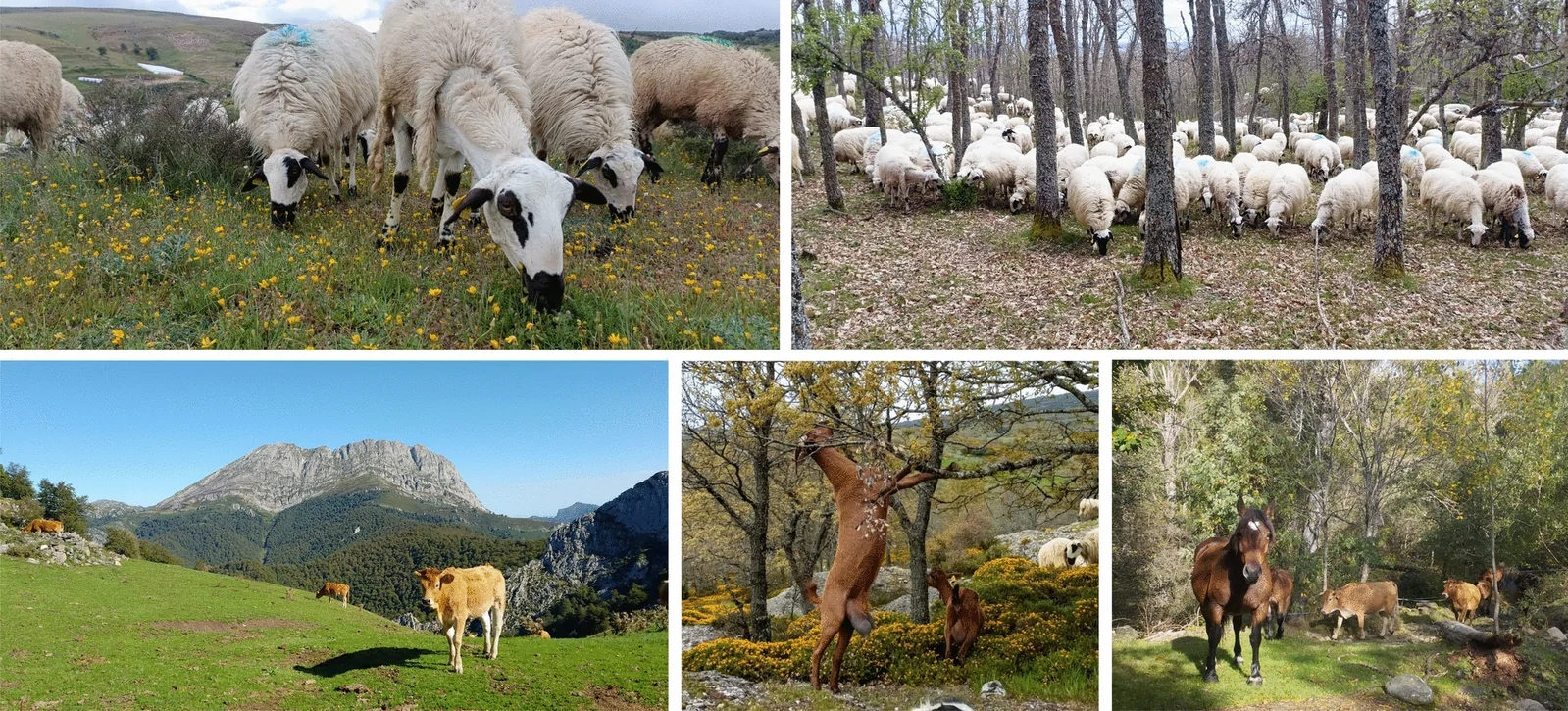
Pastoral landscapes illustrating regulating and supporting ecosystem services provided by pastoralism. Mobile herds of sheep, goats, cattle, and horses maintain open grasslands and heterogeneous vegetation structure in woodland, reducing fuel loads and wildfire risk, while promoting plant biodiversity and habitat for wildlife. Authorship (left to right and top to bottom): J.L. Fraile (1, 2, 4), F.J. Pérez-Barbería (3), A. Mendez (5)

**Fig. 2:**
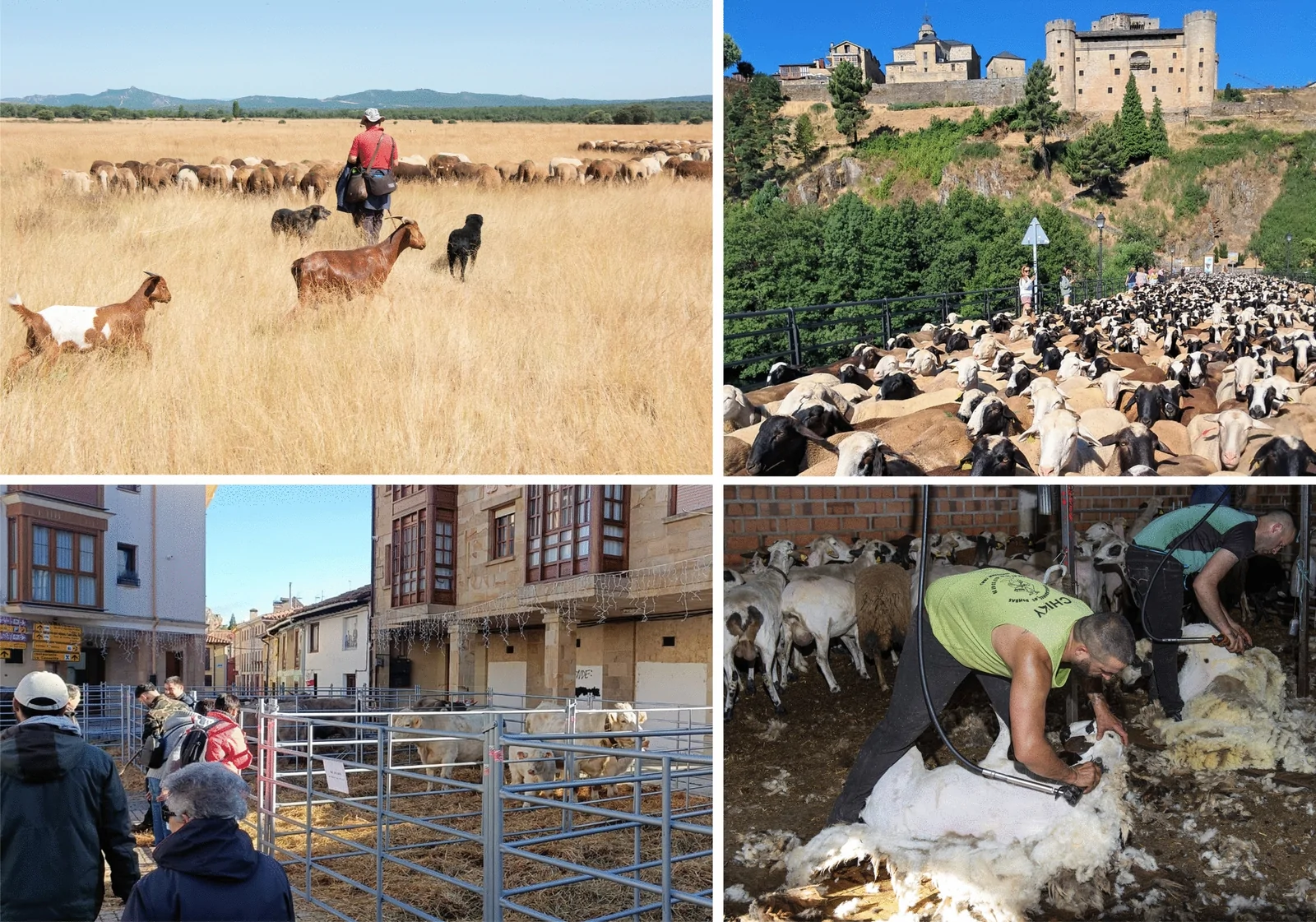
Cultural ecosystem services provided by pastoralism. Transhumant seasonal movement of livestock across landscapes, rural infrastructure, and cultural landmarks, as well as livestock shows on village streets and on-farm shearing, highlights the role of pastoralism in sustaining cultural heritage, rural livelihoods and long-term socio-ecological resilience. Authorship (left to right and top to bottom): P. Páramo (1,4), T. García (2), J.L. Fraile (3)

Pastoralism, defined here as the extensive management of livestock across rangelands, with animals feeding primarily through direct grazing on natural vegetation, has recently gained renewed attention not only for its role in food production on marginal lands, but also for the broad range of ecosystem services it provides, including regulating, supporting, and cultural services (Mancilla-Leytón et al. 2022) (Figs. 1, 2). These roles are particularly evident in Europe’s rangelands, where traditional, low-intensity livestock systems have created and maintained high nature value habitats (semi-natural grasslands, woodpastures, and mosaic landscapes rich in species diversity) as recognized in EU High Nature value farmland assessments and biodiversity (Lomba et al. 2023; Bugalho et al. 2025). Globally, this contribution has been recognized by the United Nations through the designation of 2026 as the International Year of Rangelands and Pastoralists,^1^ underscoring the critical ecological and cultural importance of these systems (Briske et al. 2025).

Despite this increasing recognition, pastoralism remains critically endangered, particularly within Europe. The survival of extensive livestock systems in the region depends largely on public subsidies, primarily through the EU Common Agricultural Policy (Kerven and Behnke 2011). Even with such support, the viability of pastoralism is increasingly undermined by a complex array of socio-economic and institutional challenges. These include low profitability of pastoral products, generational discontinuity, restricted access to pastures and transhumance routes, conflicts with wildlife, especially large carnivores, and a burdensome regulatory environment (Manzano 2021; Pérez-Barbería et al. 2024).

This precarious situation has led to a growing concern among pastoralist communities about society’s valuation of their profession. Recent studies have shown that while pastoralists express high levels of personal satisfaction with their work, they experience a sense of insufficient recognition from broader society, particularly from those in urban areas (Pérez-Barbería et al. 2024). Moreover, many pastoralists believe that societal recognition has declined over time, and they are reluctant to see their children follow in their professional footsteps. Whether this perceived limited recognition reflects an actual societal undervaluation or a miscommunication between the pastoral sector and the general public remains unverified. If unfounded, such a misperception may represent a critical gap in mutual understanding that could jeopardize the long-term survival of pastoralism and necessitate targeted communication strategies to bridge this divide (Ferguson and Bargh 2004).

The objective of the present study is to investigate the social perception of pastoralism within Spain. Specifically, I aim to identify possible mismatches between pastoralists’ expectations and societal attitudes, with the goal of informing more effective communication and policy efforts to support the sector. I hypothesize that (i) members of rural communities and older individuals will demonstrate a higher understanding and more favorable opinions of pastoralism, owing to closer cultural and historical ties to traditional livestock practices; (ii) society perceives pastoralism as more beneficial than intensive livestock systems due to its positive environmental and cultural impacts; and (iii) gender influences the valuation of pastoralism: women are likely to place greater importance on ecosystem services related to biodiversity, landscape maintenance, and animal welfare, whereas men will emphasize provisioning and production-oriented services, as supported by previous research showing gender-based differences in ecosystem service perceptions (Yang et al. 2018; Fernandez-Gimenez et al. 2021).

By shedding light on the social perception of pastoralism and the potential mismatches between pastoralists and the broader public, this study seeks to contribute to the design of more informed and socially grounded strategies for the conservation and revitalization of traditional pastoral systems.

## Study area and methods

Spain has a long-standing tradition of pastoralism, deeply rooted in its cultural heritage and rural landscapes, where transhumant and extensive livestock practices have shaped ecological and socioeconomic systems for centuries (Manzano and Casas 2010; Mangas Navas 2012; Bugalho et al. 2025). Spain continues to hold significant relevance as a pastoralist country within the EU. Eurostat data for the European Union in 2023 report around 68 million sheep and goats combined, and 74 million cattle across all 27 Member States. Spain alone accounted for approximately 23.6% of EU sheep, 23% of EU goats, and 9% of EU cattle, although the proportion of these animals managed under pastoralist systems remains poorly documented.

Pérez-Barbería et al. (2024) offer a detailed account of pastoralists’ perceptions regarding the degree of societal appreciation for pastoralism. However, it remains to be determined whether these perceptions align with the views held by the broader public. To address this question, I adopted the survey methodology described in Pérez-Barbería et al (2024) to assess public attitudes, designing a questionnaire stratified by key social variables such as age, gender, and residence (rural vs. urban), following qualitative techniques of social sciences (Rust et al. 2017). The questionnaire comprised 24 closed- and open-ended questions, structured into four thematic sections, namely, sociodemographic profile (six questions), general attitudes toward pastoralism and its services, including temporal and generational perceptions of social recognition (eight questions), social conflicts (six questions), and perceptions of the profession and its future (four questions, questionnaire in Supplementary Material). I employed a demoscopic panel to recruit participants for the online survey. Panelists are pre-registered individuals who have agreed to take part in periodic surveys and were invited to complete the questionnaire through a secure online platform. This approach allowed us to obtain responses from a demographically diverse sample aligned with the study’s target population. A total of 400 questionnaires were completed from all autonomous communities and from 48 of Spain’s 50 provinces. The questionnaire was designed for administration via digital devices. To ensure clarity and usability, a pilot test was conducted with a small group of volunteers. Based on their feedback, both the wording and structure of the questions were refined to enhance comprehension and user experience. To reduce potential biases, such as respondent priming from earlier questions or the tendency to select the first response option, both the order of questions and the sequence of answer choices were randomized for each participant. Participation was entirely voluntary, and none of the questions were mandatory; respondents could skip any question and proceed to the next without restriction. Data will be available on reasonable request.

I employed ChatGPT-5, a large language model, to assist in the classification of open-ended responses into predefined thematic groups. The model was instructed to perform an initial categorization based on semantic similarity and conceptual coherence. To ensure validity and reliability, all classifications generated by ChatGPT-5 were subsequently reviewed by the author of the study, who verified the appropriateness of the groupings and made adjustments where necessary. This combined approach leveraged the efficiency and consistency of artificial intelligence while maintaining the critical oversight of human experts, thereby enhancing both the rigor and transparency of the analytical process.

I acknowledge that relying on an online format may introduce some bias across social sectors, potentially excluding individuals without access to digital technology (Bethlehem 2010). However, digital exclusion is currently minimal in Spain. Moreover, this methodology has been successfully employed in previous studies to yield meaningful insights into public attitudes toward nature conservation (Pérez-Barbería and Gordon 2023; Pérez-Barbería et al. 2024).

### Statistical analysis

I analyzed the questionnaire data using regression models appropriate to ordered and nominal outcomes. Items measured on 5-point Likert scales (e.g., preference for rural residence; self-reported knowledge; support/concern statements; questions 4, 5, 8:15, 23, 24) were modeled with ordinal logistic regression under the proportional-odds (cumulative logit) specification. For nominal outcomes with no inherent ordering (e.g., perceived change over time in societal appreciation: “more in the past / equal / more now”; perceived appreciation by age group: “older / middle-aged / young”; questions 16, 21), I used multinomial logistic regression with the most frequent or conceptually central category as the reference.

For each ordinal model, I report coefficient estimates on the log-odds scale, their standard errors, Wald test statistics, and p values, together with cut-point (threshold) estimates separating adjacent Likert categories. Core predictors, chosen a priori, were gender (woman / man), residence (rural / urban), and age (≥ 35 / < 35); models additionally included production system type (pastoralism, extensive, and intensive) or product type (non-meat vs meat) where relevant. Where theory or descriptive patterns suggested heterogeneity, I included interaction terms (e.g., system × age; residence × gender), retaining them when jointly significant. Because our objective was to identify the main drivers of the response variables, rather than to create predictive models, I used p values to define the final model in favor to using an information criteria approach (e.g., AIC and BIC) (Murtaugh 2014). The full model was simplified using backward elimination, that is, the nonsignificant fixed-effects terms were removed one at a time following the principle of marginality: the highest order interactions were tested first and if they were significant, then the lower order effects were not tested for significance.

To aid interpretation, for each fitted model, I computed predicted probabilities for outcome categories at representative covariate profiles (e.g., urban vs rural; younger vs older; by system/product type) and as average adjusted predictions (marginal means) over the observed covariate distribution.

Prior to modeling, I inspected item distributions and coding (ensuring higher values consistently indicated stronger agreement/knowledge/preference) and verified that category orderings matched the questionnaire wording, and treated “don’t know/NA” as missing. Analyses used complete cases for the variables in each model. As sensitivity checks for key outcomes, I (i) re-specified rural exposure using an alternative acculturation indicator (rural past residence / visits to rural areas / interactions with rural residents) and (ii) compared results across model parameterizations; substantive inferences were unchanged. For ordinal models, I assessed the proportional-odds assumption with global and predictor-specific diagnostics; no violations affecting conclusions were detected.

Analyses were conducted in R software (R Core Team 2025) (v. 4.5.1) using MASS R package for ordinal models and nnet R package for multinomial models (Venables and Ripley 2002); post-estimation predictions employed marginaleffects R package (Arel-Bundock et al. 2024).

## Results

### Descriptive data on respondents and attitude to rural life

A total of 400 questionnaires were completed from all autonomous communities and from 48 of Spain’s 50 provinces (Table 1).

**Table 1:** Sociodemographic characteristics of the survey respondents

No. of questionnaires	400 (49% women, 50.7% men, 0.3% others)
Place of residence	82% urban, 16% rural
Population size of residence (no. inhabitants)	9.5% (< 5000), 6.5% (between 5000 and 30 000), 83.9% (> 30 000)
National cover (no. of provinces)	48 (number of Spanish provinces = 50)
Age (years)	Range = 17–88, interquartile range = 33–57, mean = 45
Level of education	4% basic, 32% medium, 64% high
Type of studies (high level)	51% humanities, 49% sciences

Respondents showed distinct patterns in their Likert scale ratings of rural life. Across items on living in rural areas, answers were skewed toward moderately positive options, especially among current rural residents and respondents with previous rural experience (supplementary material Tables S1, S2). Urban residents were more evenly split between attraction, ambivalence and indifference on residence preference.

### Familiarity and knowledge of livestock systems

Here, pastoralism is understood as an extensive livestock system based on animals grazing natural or semi-natural pastures directly in the field, in contrast to intensive systems based primarily on confinement and externally supplied feed. Self-reported knowledge of livestock systems varied significantly by farming system type. Pastoralism was significantly better known than both extensive and intensive livestock systems (*p* < 0.001, supplementary material Tables S3, S4), while no significant difference was found between the latter two. Predicted probabilities show that responses clustered around moderate knowledge (Likert scale 3) overall, but rural respondents were much more likely to place themselves in the higher categories: over half of rural men and around 40% of rural women selected levels 4–5, compared to about one third and one fifth among their urban counterparts, respectively. Gender differences were thus evident (*p* < 0.001, supplementary material Tables S3, S4), especially within rural areas, while age was not a significant predictor.

### Opinions on livestock system products and attitudes toward animal-derived products

Respondents held a generally favorable orientation toward animal products; however, the production system and sociodemographic factors matter: pastoralism and extensive farming were evaluated most positively, with no significant difference between them (*p* = 0.164, supplementary material Tables S5, S6). Intensive animal farming was strongly penalized against the other systems (log-odds =  − 2.6339, *p* < 0.001, supplementary material Tables S5, S6), and this rejection was especially pronounced among older respondents (log-odds =  − 0.7508, *p* = 0.0106). Rural respondents were more supportive overall than urban respondents (log-odds = 0.4789, *p* = 0.002). Old age amplifies support in general (log-odds = 0.9419, *p* < 0.001), except in the case of intensive farming, where older people are especially critical (log-odds =  − 0.7508, *p* = 0.0106). Gender did not significantly shape attitudes in the responses.

Comparing attitudes toward consuming products of animal origin (meat and non-meat) revealed an overall favorable orientation, with only a small minority expressing opposition. Predicted probabilities (supplementary material Tables S7, S8) show that support was stronger for non-meat animal products (about two-thirds rated them at the highest level on the Likert scale) than for meat products (about half rated them at the highest level). Women expressed significantly less support than men (*p* = 0.026, supplementary material Tables S7, S8), which is consistent with their lower probabilities of selecting the top category, especially for meat products. Neither rural residence nor age class significantly predicted attitudes.

### Familiarity with the pastoralist lifestyle

Most respondents showed a basic to moderate familiarity with the lifestyle of pastoralists, with nearly half selecting the midpoint of the Likert scale. Predicted probabilities (supplementary material Tables S9, S10) indicate that rural respondents were more likely to report higher familiarity than urban respondents, with almost 30% of rural residents selecting levels 4–5 compared to about 16% of urban residents (log-odds =  + 0.739, *p* = 0.005, supplementary material Tables S9, S10). Gender and age did not significantly shape the responses.

### Image of pastoralists

Most respondents clustered around moderately to strongly positive evaluations of pastoralists, with only a minority reaching the very highest level. Predicted probabilities (supplementary material Tables S11, S12) show that older individuals were much more likely to give very positive ratings (almost half selecting 5 on the Likert scale, compared to about one quarter of younger respondents), confirming their significant effect (log-odds = 1.116, *p* < 0.001, supplementary material Tables S11, S12). Neither gender nor residence significantly influenced perceptions.

### Change over time of the attitudes toward pastoralists

A multinomial model indicated a strong baseline perception of decline in social appreciation for pastoralists, with predicted probabilities (supplementary material Tables S13, S14) showing that nearly two-thirds of respondents viewed them as more appreciated in the past, while only about 15% perceived greater appreciation today (intercept terms highly significant, *p* < 0.001, supplementary material Tables S13, S14). No significant effects of gender, residence, or age were detected.

### Perceived appreciation of pastoralists across age groups

Most respondents, regardless of sociodemographic factors, believed that older people are the ones who most appreciate pastoralists, with predicted probabilities (supplementary material Tables S15, S16) showing that over 80% selected the old age sector of society, compared to about 13% for middle-aged and less than 2% for young people (log-odds =  − 2.6292, *p* < 0.001, supplementary material Tables S15, S16).

### Support for pastoral practices in land management

Most respondents tended to support the use of commons by pastoralists, while the use of fallow land (i.e., temporarily uncultivated agricultural fields), though considered important, received less support overall (log-ratio =  − 1.424, *p* < 0.001, Tables 17, 18). Predicted probabilities (supplementary material Table S17) show that support for commons increased with age, with over half of older respondents selecting the highest category (level 5 Likert scale) compared to about one third of younger respondents (log-ratio =  − 1.424, *p* = 0.001, supplementary material Tables S17, S18). In contrast, support for the use of fallow land was lower, and notably, older women showed much less support than older men (most older men selected level 4 while older women concentrated at 3; log-ratio = 0.666, *p* = 0.037, supplementary material Tables S19, S20). Residence had little influence on opinions about land management.

### Opinions on pastoral externalities and practices in shared spaces

Regarding respondents’ concerns about the use of paths and village streets by pastoralists and their livestock, older respondents were significantly more tolerant than younger ones, with predicted probabilities showing that most older participants chose the lowest concern level compared to among younger groups (log-odds =  − 0.682, *p* = 0.002, supplementary material Tables S21, S22). Rural women were more likely to be bothered than others (log-odds = 1.067, *p* = 0.048). Concerning livestock odors in villages, respondents were generally unconcerned, especially older individuals, who showed much higher probabilities at the lowest level (41% vs. 23% among younger respondents; log-odds =  − 0.837, *p* < 0.001, supplementary material Tables S23, S24). Respondents largely rejected the idea that pastoralists’ dogs pose a threat to the public, with nearly half selecting complete disagreement (supplementary material Tables S25, S26). Respondents were mostly neutral or disagreed with the idea that pastoralists interfere with farmers’ work, and older respondents were significantly less likely to endorse this view (almost half selected 1 vs. one-third of younger respondents; log-odds =  − 0.544; *p* = 0.010, supplementary material Tables S27, S28). Respondents of all sociodemographic profiles tended to agree that urban visitors to rural areas do not adapt and try to impose their way of life (supplementary material Tables S29, S30). Finally, a very small proportion (3%) reported personal conflicts with pastoralists. Most were related to: property damage by livestock, aggressive dogs, herds blocking tracks, and animal neglect.

### Perceived value toward pastoralism

The most appreciated aspects of pastoralism lifestyle in open-ended questions were: contact with nature, autonomy, animal bonding, mental wellbeing, and cultural lifestyle. The main drawbacks cited of pastoralism were: excessive workload (30%), working in harsh weather conditions (20%), sacrifice and lack of personal time, and low pay and lack of recognition.

Qualitative responses on what people liked about pastoralism emphasized: animal welfare (42.5%), naturalness (35%), product quality (22.5%), and cultural tradition and ecological value (each around 20%). Drawbacks of pastoralism highlighted: economic hardship (25%), labor intensity (22%), animal welfare concerns (15%), and environmental impact and hygiene (10%).

Respondents strongly agreed with the statement that pastoralism is disappearing. Age was the only significant predictor: older individuals were more likely to agree (*p* < 0.05). When asked whether they would like pastoralists’ children to continue in the profession, a plurality supported continuation based on freedom of choice (45.8%), followed by concern for the tradition’s disappearance (35.5%) and only a minority explicitly disagreed (4%).

## Discussion

Our results provide a nuanced picture of how society views pastoralism, and they allow a direct comparison with pastoralists’ own views as reported in recent research (Pérez-Barbería et al. 2024).

### Alignment in perceptions between the public and pastoralists

**Fig. 3:**
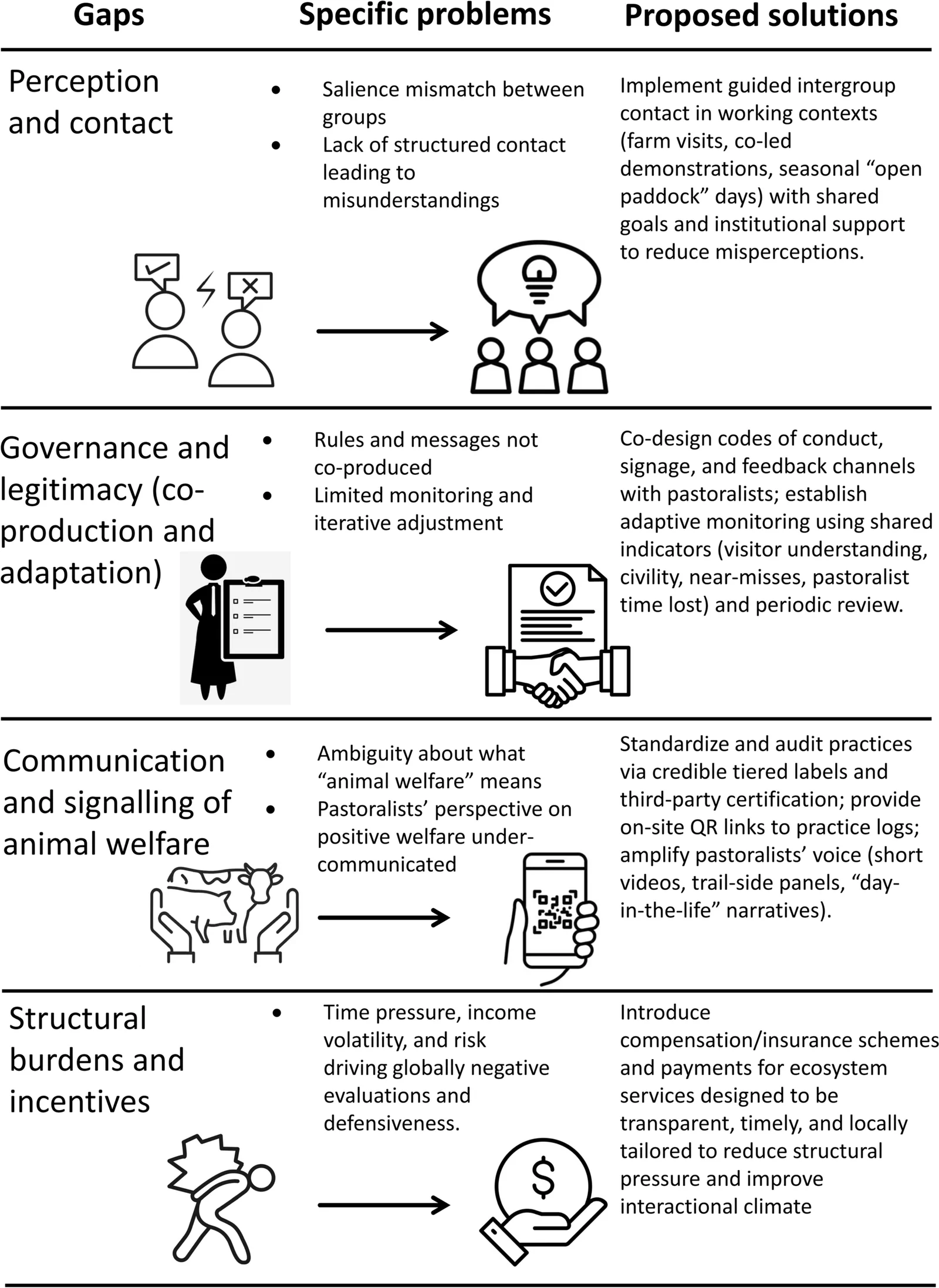
Identified gaps in the perception and valuation of pastoralism between the public and pastoralists, specific problems, and proposed interventions to improve pastoralist–public relations and pastoral governance

Our findings align with several perceptions reported by Spanish pastoralists in Pérez-Barbería et al (2024) (Fig. 3, Graphical Abstract). First, both datasets point to an age gradient in appreciation: respondents in our public sample indicated that older people value pastoralists more, echoing pastoralists’ view that esteem is concentrated among older cohorts. Second, there is shared recognition of a historical decline in social appreciation: nearly two-thirds of our respondents judged pastoralists to have been more appreciated in the past, mirroring pastoralists’ perceptions of waning societal regard (Pérez-Barbería et al. 2024). Third, it is observed a rural–urban familiarity/attitudes gradient consistent with pastoralists’ experiences: rural residence and prior rural exposure predict higher self-reported knowledge and more supportive attitudes toward pastoralism, in line with broader evidence that proximity fosters acceptance and reduces abstract risk perceptions (Boogaard et al. 2011; Byrd et al. 2017; Eijrond et al. 2023). Furthermore, land-use interfaces remain sensitive: while public support for use of commons increases with age, ambivalence about grazing fallows, especially among older women, resonates with pastoralists’ reports of ongoing frictions over shared spaces (Plieninger et al. 2015; Leroy et al. 2018; Pérez-Barbería et al. 2024). Beyond the direct comparison, our respondents drew a clear distinction between extensive/pastoral and intensive systems, with the latter viewed unfavorably, corroborating literature that links intensive units to odor, environmental, and animal welfare concerns and recognizes grazing as delivering cultural and ecological co-benefits (Hooiveld et al. 2015; Leroy et al. 2018; Alonso et al. 2020; Eijrond et al. 2022, 2023).

### Mismatches in perceptions between the public and pastoralists

Perceived urban intolerance to pastoral practices diverges: Pastoralists emphasize nuisance externalities such as dirt, odors, noise, and dog threats, particularly among urban visitors, as key drivers of disfavor, whereas our public sample expressed low concern about livestock odors in villages and largely rejected the idea that pastoralists’ dogs pose a public threat; tolerance was highest among older respondents (Pérez-Barbería et al. 2024). This pattern suggests a communication/salience gap in which episodic, visible conflicts weigh heavily for pastoralists but are not widely experienced by the median citizen (Hooiveld et al. 2015; Eijrond et al. 2019, 2023).

A second divergence concerns the valuation of pastoral products. Pastoralists perceived urban society as undervaluing these products (Pérez-Barbería et al. 2024); yet, our data show strong public support for animal-derived products from pastoral and extensive systems relative to intensive ones, consistent with European evidence linking pasture-based systems to higher perceived animal welfare and cultural ecosystem benefits, even if this does not always translate into purchasing behavior or willingness-to-pay (Boogaard et al. 2011; Alonso et al. 2020; Cei et al. 2023).

Open-ended responses further reveal a salience asymmetry. Pastoralists prioritize intrinsic job rewards (autonomy, working in nature) alongside structural burdens (time demands, bureaucracy, and income volatility) and working on harsh weather conditions; whereas the public foregrounds ethical and product attributes (animal welfare, naturalness, and quality) and negative externalities (environmental/hygiene), while acknowledging labor intensity. Thus, both groups recognize the demanding nature of pastoral work, but frame animal welfare differently: the public cites it both as a benefit of pastoralism and as an area of concern, whereas pastoralists emphasize welfare through care and human–animal bonding (Leroy et al. 2018; Pérez-Barbería et al. 2024).

### Explaining perceptual differences between the public and pastoralists

Differences in perception between the public and pastoralists may be shaped by how frequently each group is exposed to conflict. General cognitive processes and asymmetric stakes suggest that the relationship between exposure to pastoralist–public conflicts and negative evaluations is non-linear (Puri et al. 2024; Sundh 2024). Among pastoralists, daily exposure can dampen per-incident negativity via habituation or desensitization: repeated stressors elicit smaller affective responses over time, an effect documented in the literature on community violence and recurrent workplace aggression (Gaylord-Harden et al. 2017; Yragui et al. 2017). At the same time, cumulative exposure entails material and psychological costs that can worsen global evaluations of the conflict phenomenon, even if any single episode feels routine (Kansky et al. 2014; Dressel et al. 2015). Pastoralists may thus appraise a given incident as “part of the job” while holding more negative overall attitudes due to accumulated harm.

Taken together, our results suggest that pastoralists may display lower per-incident reactivity but more negative global evaluations owing to accumulated costs, whereas the public may display higher per-incident negativity (availability, affect, and negativity biases) yet more positive aggregate attitudes when not directly affected. This pattern aligns with findings from adjacent conflict domains (Baumeister et al. 2001; Kansky et al. 2014; Dressel et al. 2015).

### Bridging the salience gap between the public and pastoralists

Closing the salience gap requires interventions that (i) create structured, repeated contact, (ii) co-produce rules and messages for shared spaces, (iii) turn animal welfare and product claims into trusted standards, and (iv) ease structural burdens on herders.

Structured intergroup contact in working settings (e.g., guided farm and herding visits, short demonstrations on trails, or seasonal “open paddock” days) can correct misperceptions by exposing visitors to routine care practices. Meta-analyses show that such contact, when organized around common goals and cooperation and backed by institutions, reliably reduces prejudice (Pettigrew and Tropp 2006, 2008; Tropp et al. 2022).

Co-produced “rules of engagement” and shared messaging stabilize expectations. Joint codes of conduct, signage, and feedback channels enhance perceived procedural fairness and social acceptability; in rural areas, this can mean small stewardship committees that set micro-rules for trails, streets and commons, complemented by concise good-neighbor agreements and simple mediation of recurrent disputes (Ostrom 1990; Treves et al. 2006).

Because the public foregrounds animal welfare and “naturalness”, practices need to be concrete and communicable at the farm-neighbor boundary. Translating extensive-system practices into recognized third-party standards and clear on-site cues can turn diffuse concern into informed support, provided labels are understood and certifiers trusted (Grunert et al. 2014, 2018; Alonso et al. 2020). Yet, visible logos or project signage may clash with locally valued “good farmer” identities and reconfigure symbolic capital in tight rural communities, sometimes triggering envy or suspicion (Bourdieu 1990; Burton and Wilson 2006).

Narrative interventions should center pastoralists’ own language of positive welfare, which emphasizes proactive care and opportunities for natural behavior rather than the mere absence of harm. Short videos, trail-side panels, and “day-in-the-life” stories can present these framings and recognize herders’ expertise (Muhammad et al. 2024).

Finally, because pastoralists’ globally negative evaluations are rooted in chronic workload and lack of personal time (‘time pressure’), income volatility and risk, institutional supports are needed to reduce defensiveness and enable cooperative norms. Relevant measures include rapid, transparent compensation or insurance for accidental damage, payments for ecosystem services linked to grazing-dependent biodiversity and fire-risk reduction, and modest grants for shared infrastructure. Although this study did not directly evaluate public attitudes toward subsidies, support schemes may create tension when they are perceived as opaque or inequitable, which is why compensation and payments for ecosystem services should be transparent, timely, and locally tailored. Boundary organizations such as cooperatives or parish councils can broker between pastoralists, residents and regulators and convene periodic joint activities. Evidence suggests that well-designed, co-produced schemes improve wellbeing and acceptance without systematically crowding out intrinsic stewardship (Pretty and Ward 2001; Cash et al. 2003; Reed et al. 2017; Shen et al. 2022; Vorlaufer et al. 2023).

## Implications

Our findings indicate a latent social license for pastoralism in Spain. The public, particularly older and rural respondents, tend to value pastoralism and other extensive livestock systems more highly than intensive systems and express relatively low concern about associated externalities. At the same time, pastoralists underestimate this acceptance, especially in urban contexts, and perceive a sharper loss of social esteem. These results suggest that policies and governance arrangements should both recognize and nurture existing public support and directly address the structural and symbolic burdens that fuel pastoralists’ pessimism, for example through targeted communication, co-designed rules for shared spaces and mechanisms that reduce everyday frictions. An important caveat is that social license for pastoralism appears concentrated among older and rural groups and may not persist through generational turnover. Its maintenance will likely require active renewal through education, structured pastoralist–citizen contact, clearer communication of animal welfare and ecosystem service benefits, and institutional support.

## Supplementary Information

Below is the link to the electronic supplementary material.

Supplementary file1 (PDF 672 KB)

## Footnotes

1
https://iyrp.info/.
