# Supplementary material for: Public–pastoralist perception gaps in valuing pastoralism: Implications for its sustainability
Source: Ambio. 2026 Apr 29;55(12):2949–60. doi: 10.1007/s13280-026-02401-2 (PMC13731543; doi:10.1007/s13280-026-02401-2)
Supplement: Supplementary file 1 [file 13280_2026_2401_MOESM1_ESM.pdf]

Supplementary Information

Title: Public–pastoralist perception gaps in valuing pastoralism: Implications for its sustainability
Journal: Ambio

Authors: F. Javier Pérez-Barbería*
* Corresponding author. Affiliation: Instituto Mixto de Investigación en Biodiversidad (CSIC – Universidad de Oviedo – Principado de Asturias). Universidad de Oviedo-Campus de Mieres, Edificio de Investigación, 33600 Mieres, Asturias, Spain.
E-mail: j.perez.barberia@csic.es, j.perezbarberia@gmail.com

Questionnaire

FILTER_1. Which two items of clothing are *not* worn on the upper part of the body?
Items shown in random order

1. T-shirt
2. Jacket
3. Socks
4. Shoe
5. None of these *(do not rotate, exclusive answer)*

If respondents do not choose options 3 and 4, screen out.

P1. How old are you?
Show as a drop-down list. If the person is under 16 years old, screen out.

P2. You are…

1. Male
2. Female
3. Another option / Prefer to self-describe

P2b. In which province do you live?
Show as a drop-down list.

P4. Rural areas are places where few people live. They are usually small towns and villages where many people work in farming or raising animals. They offer fewer services (shops, hospitals, schools, etc.) than cities do.

Do you live in a rural area?

1. Yes
2. No
3. I'm not sure

P4d. What is the main type of economic activity in the place where you live?

1. Primary sector (farming, livestock, fishing, etc.)
2. Secondary sector (industry, construction, energy production)
3. Tertiary sector (commerce, tourism, education, finance, etc.)
4. I'm not sure

---

*Ask only if they did **not** choose option 1 in P4.*

P4b. In the past, have you ever lived in rural areas or spent long periods of time there?

1. Yes
2. No
3. I'm not sure

---

*Ask only if they did **not** choose option 1 in P4.*

P4c. How often do you usually visit rural areas?

1. Never
2. Rarely
3. Sometimes
4. Often
5. Very often

---

*Ask only if they did **not** choose option 1 in P4.*

P5. Whether it is face to face, by phone or on the internet…
How often do you usually talk with people who live in rural areas?

1. Never

2. Rarely
3. Sometimes
4. Often
5. Very often

P5b. What score would you give rural areas as places to live and to enjoy your free time?

Show a scale from 0 to 5, plus “I don’t know”.
Label the ends of the scale as “I don’t like them at all” (0) and “I like them a lot” (5).

Items shown in random order:

1. As a place to live
2. As a place to enjoy my free time

P13a. Let’s change topic now. As you know, people often eat meat from animals.

Are you in favour or against people eating meat?

1. Strongly in favour
2. Quite in favour
3. Neither in favour nor against
4. Quite against
5. Strongly against

P13b. There are other animal products that do not require killing animals. Milk, cheese and eggs are some examples.

Are you in favour or against people consuming this type of product?

1. Strongly in favour
2. Quite in favour
3. Neither in favour nor against
4. Quite against
5. Strongly against

P13c. How well do you know the following ways of raising and caring for animals?

Answer options:

- I don’t know it

- I’ve heard of it, but I don’t know what it is
- I know a little about it
- I know quite a bit about it
- I know it very well

Items shown in random order:

1. Extensive livestock farming
2. Intensive livestock farming
3. Traditional pastoralism / herding

Next, we will show you 3 sentences related to the ways of raising and caring for animals that we mentioned above. Please tell us how much you agree with each one.
Rotate these questions.

P9d. “I have a good opinion of products (milk, meat, cheese, etc.) that come from extensive livestock farming (animals raised outdoors, on large areas of land, feeding mainly on natural pastures).”

1. Strongly agree
2. Somewhat agree
3. Neither agree nor disagree
4. Somewhat disagree
5. Strongly disagree
6. I’m not sure

P9. “I have a good opinion of products (milk, meat, cheese, etc.) that come from intensive livestock farming (animals raised in closed or small spaces, mainly fed with feed and often supported by technologies to increase production quickly).”

1. Strongly agree
2. Somewhat agree
3. Neither agree nor disagree
4. Somewhat disagree
5. Strongly disagree
6. I’m not sure

P8. “I have a good opinion of products (milk, meat, cheese, etc.) that come from traditional pastoralism (animals raised outdoors, guided by shepherds near the farm so they can feed on natural pastures, or sometimes moved long distances on seasonal routes).”

1. Strongly agree
2. Somewhat agree

3. Neither agree nor disagree
4. Somewhat disagree
5. Strongly disagree
6. I’m not sure

In the following questions, we would like to know your opinion about traditional pastoralism.

Traditional pastoralism is a way of caring for and feeding livestock (such as sheep, goats or cattle) in which the animals are guided on foot by shepherds so that they can eat directly from natural pastures.

Rotate P19 and P20.

P19. What do you like most about traditional pastoralism?
Open text box.

P20. What do you like least about traditional pastoralism?
Open text box.

A shepherd is the person who best represents traditional pastoralism. They look after and guide a group of animals while they graze freely in the countryside. They make sure the animals eat properly and protect them from danger.

Rotate P22 and P23.

P22. What do you think is the best thing about being a shepherd?
Open text box.

P23. What do you think is the worst thing about being a shepherd?
Open text box.

P23b. How familiar are you with the lifestyle of shepherds?

1. I know their lifestyle very well
2. I know their lifestyle quite well
3. I have a basic idea of their lifestyle
4. I’ve heard about it, but I know very little
5. I have never heard about the lifestyle of shepherds

P23c. What is your overall opinion of traditional shepherds?

Show a scale from 0 to 5, where 0 is “very negative” and 5 is “very positive”.
Add the option “I don’t know”.

P16. In your opinion…

1. A few years ago, shepherds were more appreciated than they are now
2. Nowadays shepherds are more appreciated than a few years ago
3. It has not changed: the appreciation for shepherds is the same
4. I’m not sure

P16. In your opinion, who appreciates shepherds the most?

1. Older people
2. Middle-aged people
3. Young people
4. I’m not sure

P21. Would you like the sons and daughters of shepherds to continue working as shepherds?

1. Yes
2. No
3. I don’t mind / It’s all the same to me

P21b. In the previous question you said: [answer given in P21]. Could you explain your reasons?
Open text box.

P24. How much do you agree or disagree with the following sentence?

“Traditional pastoralism and the profession of shepherd are slowly disappearing.”

1. Strongly agree
2. Somewhat agree
3. Neither agree nor disagree
4. Somewhat disagree

5. Strongly disagree
6. I'm not sure

Next, we will show you 6 sentences. Please tell us how much you agree with each one. Rotate the following block of questions.

P10a. "It bothers me that in some villages livestock (such as cows, sheep, goats or donkeys) walk along paths or streets, even if they are accompanied by shepherds or farmers."

1. Strongly agree
2. Somewhat agree
3. Neither agree nor disagree
4. Somewhat disagree
5. Strongly disagree
6. I'm not sure

P10b. "The smells from animals in traditional pastoralism bother me when I'm in villages."

1. Strongly agree
2. Somewhat agree
3. Neither agree nor disagree
4. Somewhat disagree
5. Strongly disagree
6. I'm not sure

P11. "People from cities who visit rural areas don't adapt and try to impose their way of life."

1. Strongly agree
2. Somewhat agree
3. Neither agree nor disagree
4. Somewhat disagree
5. Strongly disagree
6. I'm not sure

P12. "When I am walking on paths in rural areas, I feel threatened by shepherds' dogs."

1. Strongly agree
2. Somewhat agree

3. Neither agree nor disagree
4. Somewhat disagree
5. Strongly disagree
6. I’m not sure

P14. “Shepherds make the work of farmers and other people who work the land more difficult.”

1. Strongly agree
2. Somewhat agree
3. Neither agree nor disagree
4. Somewhat disagree
5. Strongly disagree
6. I’m not sure

P15a. “I think it is fine for shepherds to use common or communal land to feed their animals.”

1. Strongly agree
2. Somewhat agree
3. Neither agree nor disagree
4. Somewhat disagree
5. Strongly disagree
6. I’m not sure

P15b. “I think it is fine for shepherds to use what is left in the fields after the harvest, such as leftover plant material, to feed their animals.”

1. Strongly agree
2. Somewhat agree
3. Neither agree nor disagree
4. Somewhat disagree
5. Strongly disagree
6. I’m not sure

P14. Have you ever had a confrontation, argument or conflict with a shepherd?

1. Yes
2. No

We are almost finished. We now have just a few questions that will help us to prepare the statistics. Once again, thank you very much for your help.

P6. What is the highest level of education you have completed?

1. Primary education
2. Secondary education
3. Higher education (university or equivalent)

Only if P6 = 6 or 7.

P6b. Which area of knowledge best matches your university studies?
Items shown in random order.

1. Sciences
2. Arts and Humanities

P3b. What is your current employment situation?

1. Self-employed
2. Employed by someone else
3. Unemployed
4. Student
5. Retired
6. I do unpaid household work / care work
7. I have a permanent disability
8. Other situation

Only if P3b = 1 or 2.

P3. Which of the following activities best matches your job?

1. Agriculture, livestock, forestry and fishing
2. Extractive industries (stone and mineral extraction)
3. Manufacturing industry, production of goods
4. Supply of electricity, gas and air conditioning
5. Water supply and waste management
6. Construction
7. Commerce / Retail and wholesale trade
8. Transport and storage
9. Hospitality (bars, restaurants, hotels, etc.)
10. Content creation: television, radio, press, etc.

11. Telecommunications and computer programming
12. Financial and insurance activities
13. Real-estate activities
14. Scientific and technical activities
15. Administrative activities
16. Public administration and defence
17. Education
18. Health care and social services
19. Artistic, sports and entertainment activities
20. Domestic work: caregiver, house cleaning, etc.
21. Activities of organisations and extra-territorial bodies
22. Other

C1. MANY THANKS FOR YOUR COLLABORATION

Have you had any problem while filling out this survey, or is there anything you would like to tell us about it before finishing?
Open question.

Tables

Table 1. Predicted probabilities for preference to live in a rural residence (model: Table 2). Only total and statistically significant predictors are shown. Likert scale from 1 (“I don’t like it at all”) to 5 (“I like it a lot”).

Likert-scale Residence	1	2	3	4	5
Urban	0.0585	0.105	0.344	0.267	0.225
Rural	0.0143	0.0296	0.152	0.252	0.553
Total	0.0512	0.0929	0.3123	0.2645	0.279

Table 2. Ordinal logistic regression results on preferences for living in a rural residence by gender (reference: man), residence (reference: urban) and age (reference: <35 years old) of respondent. Thresholds between Likert-scale categories from 1 “I don’t like it at all” *to 5* “I like it a lot”.

Predictor / Threshold	Coefficient (log-odds)	Std. Error	t value	p value
Gender (woman)	-0.2381	0.1923	-1.2382	0.2167
Residence (rural)	1.4661	0.2632	5.5702	2.54e-08
Age (≥35 years)	0.2534	0.2083	1.2163	0.2239
1|2	-2.7364	0.3036	-9.0141	1.99e-19
2|3	-1.5837	0.2453	-6.4570	1.07e-10
3|4	0.0891	0.2248	0.3964	0.6918
4|5	1.3021	0.2356	5.5267	3.26e-08

Table 3. Ordinal logistic regression results on knowledge of animal farming systems (reference: extensive system) by gender (reference: man), residence (reference: urban) and age (reference: <35 years old) of respondent. Thresholds between Likert-scale categories from 1 “I don’t know it at all” *to 5* “I know it very well”.

Predictor / Threshold	Coefficient (log-odds)	Std. Error	t value	p value
Intensive system	0.0112	0.1316	0.0854	0.932
Pastroralism system	0.8319	0.1328	6.2651	3.73e-10
Gender (woman)	-0.5373	0.1119	-4.8030	1.56e-06
Residence (rural)	0.9803	0.1526	6.4243	1.32e-10
Age (≥35 years)	0.1124	0.1203	0.9342	0.350
1 | 2	-1.8444	0.1645	-11.2099	3.65e-29
2 | 3	-0.8124	0.1534	-5.2948	1.19e-07
3 | 4	1.1721	0.1558	7.5245	5.29e-14
4 | 5	2.8436	0.1807	15.7365	8.50e-56

Table 4. Predicted probabilities for knowledge of animal farming systems (model: Table 3). Only total and statistically significant predictors are shown. Likert scale from 1 “I don’t know it at all” *to 5* “I know it very well”.

Likert-scale		1	2	3	4	5
Residence	Gender					
Urban	Man	0.103	0.138	0.443	0.233	0.083
No	Woman	0.167	0.188	0.434	0.162	0.0493
Rural	Man	0.042	0.0668	0.35	0.351	0.19
Rural	Woman	0.0719	0.105	0.418	0.286	0.118
Total		0.1229	0.1504	0.4289	0.2171	0.0808

Table 5. Predicted probabilities for preferences for animal products from different farming systems (model: Table 6). Only total and statistically significant predictors are shown. Likert-scale categories from 1 “I am completely against it” *to 5* “I am completely in favor of it”.

Likert-scale			1	2	3	4	5
Residence	Age (years)	Farming type					
Urban	<35	Pastoralism	0.0305	0.0486	0.186	0.267	0.467
Urban	<35	Intensive	0.305	0.24	0.29	0.107	0.0593
Urban	<35	Extensive	0.042	0.0648	0.228	0.279	0.387
Urban	>=35	Pastoralism	0.0117	0.0197	0.0886	0.181	0.699
Urban	>=35	Intensive	0.259	0.229	0.312	0.126	0.0732
Urban	>=35	Extensive	0.0196	0.0321	0.135	0.233	0.581
Rural	<35	Pastoralism	0.019	0.0313	0.132	0.23	0.587
Rural	<35	Intensive	0.213	0.212	0.332	0.151	0.0929
Rural	<35	Extensive	0.0263	0.0423	0.168	0.258	0.506
Rural	>=35	Pastoralism	0.0072	0.0122	0.0575	0.131	0.792
Rural	>=35	Intensive	0.176	0.192	0.342	0.175	0.114
Rural	>=35	Extensive	0.012	0.0202	0.0906	0.184	0.694
Total		Pastoralism	0.0162	0.0266	0.112	0.2	0.646
Total		Intensive	0.259	0.227	0.311	0.128	0.0753
Total		Extensive	0.0246	0.0394	0.154	0.24	0.541

Table 6. Ordinal logistic regression results on preferences for animal products from different farming systems (reference: pastoralism) by gender (reference: man), residence (reference: urban) and age (reference: <35 years old) of respondent. Thresholds between Likert-scale categories from 1 “I am completely against it” *to 5* “I am completely in favor of it”.

Predictor / Threshold	Coefficient (log-odds)	Std. Error	t value	p value
Residence (rural)	0.4789	0.1560	3.0710	0.00213
Gender (woman)	-0.1202	0.1165	-1.0326	0.302
Intensive system	-2.6339	0.2508	-10.5030	8.37e-26
Extensive system	-0.3304	0.2375	-1.3912	0.164
Age (≥35 years)	0.9419	0.2173	4.3352	1.46e-05

Intensive x Age (≥35 years)	-0.7508	0.2939	-2.5547	0.0106
Extensive x Age (≥35 years)	-0.1877	0.2967	-0.6327	0.527
1|2	-3.5449	0.2277	-15.5715	1.14e-54
2|3	-2.5408	0.2128	-11.9376	7.54e-33
3|4	-1.1042	0.1962	-5.6266	1.84e-08
4|5	0.0452	0.1921	0.2355	0.814

Table 7. Predicted probabilities for preferences for type of animal products (model: Table 8). Only total and statistically significant predictors are shown. Likert-scale categories from 1 “I am completely against it” *to 5* “I am completely in favor of it”.

Likert-scale		1	2	3	4	5
Residence	Product					
Man	Meat	0.0102	0.0292	0.196	0.228	0.536
Man	Animal non-meat	0.0051	0.0148	0.113	0.167	0.7
Women	Meat	0.0137	0.0386	0.241	0.244	0.463
Women	Animal non-meat	0.0068	0.0197	0.144	0.195	0.635
Total	Meat	0.012	0.0339	0.219	0.236	0.499
Total	Animal non-meat	0.0059	0.0173	0.128	0.181	0.667
Total	Meat	0.012	0.0339	0.219	0.236	0.499

Table 8. Ordinal logistic regression results on preferences for animal products (reference: meat) by gender (reference: man), residence (reference: urban) and age (reference: <35 years old) of respondent. Thresholds between Likert-scale categories from 1 “I am completely against it” *to 5* “I am completely in favor of it”.

Predictor / Threshold	Coefficient (log-odds)	Std. Error	t value	p value

Non-meat animal product	0.7043	0.1425	4.9437	7.66e-07
Residence (rural)	-0.1226	0.1928	-0.6359	0.525
Gender (woman)	-0.3270	0.1473	-2.2194	0.0265
Age (≥35 years)	-0.1209	0.1602	-0.7549	0.450
1|2	-4.6963	0.4166	-11.2742	1.76e-29
2|3	-3.3157	0.2605	-12.7265	4.21e-37
3|4	-1.2978	0.1918	-6.7682	1.30e-11
4|5	-0.2659	0.1849	-1.4382	0.150

Table 9. Predicted probabilities for familiarity with the pastoral lifestyle (model: Table 10). Only total and statistically significant predictors are shown. Likert-scale categories from 1 "I don't know it at all" *to 5* "I know it very well".

Likert-scale Residence	1	2	3	4	5
Urban	0.0698	0.285	0.481	0.107	0.0574
Rural	0.0349	0.175	0.5	0.177	0.112
Total	0.0641	0.2672	0.4839	0.1185	0.0664

Table 10. Ordinal logistic regression results on familiarity with the pastoral lifestyle by gender (reference: man), residence (reference: urban) and age (reference: <35 years old) of respondent. Thresholds between Likert-scale categories from 1 "I don't know it at all" *to 5* "I know it very well".

Predictor / Threshold	Coefficient (log-odds)	Std. Error	t value	p value

Gender (woman)	-0.2527	0.1969	-1.2829	0.200
Residence (rural)	0.7393	0.2632	2.8089	0.00497
Age (≥35 years)	0.2566	0.2151	1.1927	0.233
1 | 2	-2.5533	0.2920	-8.7448	2.23e-18
2 | 3	-0.5496	0.2339	-2.3497	0.0188
3 | 4	1.6915	0.2492	6.7871	1.14e-11
4 | 5	2.8683	0.2950	9.7222	2.43e-22

Table 11. Predicted probabilities for positive perception of traditional herders´ image (model: Table 12). Only total and statistically significant predictors are shown. Likert-scale categories from 1 “Very negative perception of herders´ image” *to 5* “Very positive perception of herders´ image”.

Likert-scale<br>Age	1	2	3	4	5
<35	0.0049	0.0241	0.315	0.423	0.233
>=35	0.0015	0.0078	0.133	0.369	0.488
Total	0.0026	0.0127	0.1881	0.3853	0.4113

Table 12. Ordinal logistic regression results on positive perception of traditional herders´ image by gender (reference: man), residence (reference: urban) and age (reference: <35 years old) of respondent. Thresholds between Likert-scale categories from 1 “Very negative perception of herders´ image” *to 5* “Very positive perception of herders´ image”.

Predictor / Threshold	Coefficient (log-odds)	Std. Error	t value	p value
Gender (woman)	-0.1556095	0.1981487	-0.7853168	4.32E-01
Residence (rural)	0.2206883	0.2626461	0.8402497	4.01E-01
Age (≥35 years)	1.1156118	0.2160593	5.1634519	2.42E-07
Muy negativa 1|2	-5.3912708	1.0186199	-5.2927211	1.21E-07

2|3	-3.5828286	0.4524468	-7.9187844	2.40E-15
3|4	-0.7140536	0.2302986	-3.1005553	1.93E-03
4|Muy positiva 5	1.1297616	0.2359591	4.787956	1.68E-06

Table 13. Predicted probabilities for perceptions of change over time in societal appreciation of traditional herders (model: Table 14). Only the overall total and statistically significant predictors are shown. Categories: Before "More appreciated in the past," Equally "Appreciation unchanged over time," Now "More appreciated at present."

	Before	Equally	Now
Probability	0.6364	0.217	0.1466

Table 14. Multinomial logistic regression results examining the perceived changes across time in the appreciation of society to traditional herders by gender (reference: man), residence (reference: urban) and age (reference: <35 years old) of respondent. Outcome: more appreciated now (now), equally appreciated before and now (equally), reference outcome (more appreciated before). Model fit indices include residual deviance and AIC.

Outcome	Predictor	Coefficient (log-odds)	Std. Error	p value
equally	(Intercept)	-1.4128	0.3342	2.23e-05
equally	Gender (woman)	0.4594	0.2804	0.1014
equally	Residence (rural)	-0.3732	0.4013	0.352
equally	Age (≥35 years)	0.2121	0.3075	0.490
now	(Intercept)	-1.6372	0.3810	1.73e-05
now	Gender (woman)	0.1336	0.3235	0.680

now	Residence (rural)	-0.0828	0.4277	0.846
now	Age (≥35 years)	0.1673	0.3588	0.641

Residual Deviance: 610.12

AIC: 626.12

Table 15. Predicted probabilities for perceived appreciation of different age classes of society to traditional herders (model: Table 16). Only the overall total and statistically significant predictors are shown. Categories: old "old age sector of society", middle "middle age sector of society", young "young age sector of society".

Age	old	middle	young
<35	0.882	0.1	0.0182
>=35	0.831	0.151	0.0178
Total	0.8478	0.1343	0.0179

Table 16. Multinomial logistic regression results examining the perceived appreciation of different age classes of society to traditional herders by gender (reference: man), residence (reference: urban) and age (reference: <35 years old) of respondent. Outcome: more appreciated by people of middle age (middle), more appreciated by people of young age (young), reference outcome (more appreciated by people of old age). Model fit indices include residual deviance and AIC.

Outcome	Predictor	Coefficient	Std. Error	p value
middle	(Intercept)	-2.6292	0.4298	9.50e-10
middle	Gender (woman)	0.6564	0.3415	0.0546
middle	Residence (rural)	-0.2694	0.5072	0.595
midle	Age (≥35 years)	0.6425	0.3825	0.0930

young	(Intercept)	-3.7458	0.9371	6.40e-05
young	Gender (woman)	-0.6722	0.9147	0.462
young	Residence (rural)	0.9917	0.8872	0.264
young	Age (≥35 years)	-0.0988	0.9192	0.914
Residual Deviance: 314.77				
AIC: 330.77				

Table 17. Predicted probabilities for support for traditional herders using commons (model: Table 18). Only total and statistically significant predictors are shown. Likert-scale categories from 1 “I am completely against it” *to 5* “I am completely in favor of it”.

Likert-scale<br>Age	1	2	3	4	5
<35	0.0396	0.0384	0.294	0.269	0.359
>=35	0.0194	0.0196	0.182	0.24	0.538
Total	0.0255	0.0253	0.216	0.2489	0.4843

Table 18. Ordinal logistic regression results on support for traditional herders using commons, by gender (reference: man), residence (reference: urban) and age (reference: <35 years old) of respondent. Thresholds between Likert-scale categories from 1 “I am completely against it” *to 5* “I am completely in favor of it”.

Predictor / Threshold	Coefficient (log-odds)	Std. Error	t value	p value
Gender (woman)	-0.2362	0.1979	-1.1937	0.233
Residence (rural)	-0.0425	0.2563	-0.1660	0.868

Age (≥35 years)	0.6657	0.2103	3.1651	0.00155
1|2	-3.3690	0.3754	-8.9753	2.83e-19
2|3	-2.6493	0.3016	-8.7833	1.59e-18
3|4	-0.7005	0.2271	-3.0845	0.00204
4|5	0.4062	0.2257	1.8001	0.0718

Table 19. Ordinal logistic regression results on support for traditional herders using fallows, by gender (reference: man), residence (reference: urban) and age (reference: <35 years old) of respondent. Thresholds between Likert-scale categories from 1 “I am completely against it” *to 5* “I am completely in favor of it”.

Predictor / Threshold	Coefficient (log-odds)	Std. Error	t value	p value
Gender (woman)	0.4607	0.5474	0.8416	0.400
Age (≥35 years)	0.2986	0.5154	0.5795	0.562
Residence (rural)	0.7074	0.4475	1.5809	0.114
Gender (woman):Age (≥35 years)	-1.4843	0.7133	-2.0809	0.0374
1|2	-2.6985	0.5424	-4.9752	6.52e-07
2|3	-1.9004	0.4763	-3.9902	6.60e-05
3|4	0.0469	0.4321	0.1085	0.914
4| 5	30.4906	0.4321	70.5694	0.000

Table 20. Predicted probabilities for support for traditional herders using fallows (model: Table 19). Only total and statistically significant predictors are shown. Likert-scale categories from 1 “I am completely against it” *to 5* “I am completely in favor of it”.

Likert-scale		1	2	3	4	5
Gender	Age					
Man	<35	0.056	0.06	0.357	0.527	0
Man	>=35	0.0436	0.0481	0.318	0.59	0
Woman	<35	0.0368	0.0413	0.29	0.631	0
Woman	>=35	0.115	0.109	0.441	0.335	0
Total		0.0642	0.0654	0.3516	0.5188	0

Table 21. Predicted probabilities for respondents' concerns about the use of paths and village streets by herders and their livestock (model: Table 22). Only total and statistically significant predictors are shown. Likert-scale categories from 1 "Not concerned at all" *to 5* "Very concerned".

Likert-scale			1	2	3	4	5
Residence	Gender	Age					
Urban	Hombre	<35	0.467	0.236	0.198	0.0636	0.0361
Urban	Hombre	>=35	0.634	0.19	0.123	0.0344	0.0186
Urban	Mujer	<35	0.475	0.234	0.194	0.0619	0.0351
Urban	Mujer	>=35	0.641	0.187	0.12	0.0335	0.0181
Rural	Hombre	<35	0.522	0.224	0.172	0.0525	0.0293
Rural	Hombre	>=35	0.683	0.17	0.104	0.0281	0.015
Rural	Mujer	<35	0.278	0.231	0.289	0.123	0.0785
Rural	Mujer	>=35	0.433	0.24	0.214	0.0716	0.0413
Total			0.5769	0.2025	0.1492	0.0457	0.0257

Table 22. Ordinal logistic regression results on respondents' concerns about the use of paths and village streets by herders and their livestock, by gender (reference: man), residence (reference: urban) and age (reference: <35 years old) of respondent. Thresholds between Likert-scale categories from 1 "Not concerned at all" *to 5* "Very concerned".

Predictor / Threshold	Coefficient (log-odds)	Std. Error	t value	p value
Gender (woman)	-0.0302417	0.2247323	-1.35E-01	8.93E-01
Residence (rural)	-0.2186285	0.398683	-5.48E-01	5.83E-01
Age (≥35 years)	-0.6822542	0.2207505	-3.09E+00	2.00E-03

Gender:Residence	1.06987015	0.5414857	1.98E+00	4.82E-02
1|2	-0.1320068	0.2404259	-5.49E-01	5.83E-01
2|3	0.85968988	0.2443069	3.52E+00	4.33E-04
3|4	2.20016395	0.2862756	7.69E+00	1.52E-14
4|5	3.28330118	0.3821864	8.5908373	8.63E-18

Table 23. Ordinal logistic regression results on respondents' concerns about livestock odours in villages, by gender (reference: man), residence (reference: urban) and age (reference: <35 years old) of respondent. Thresholds between Likert-scale categories from 1 "Not concerned at all" *to 5* "Very concerned".

Predictor / Threshold	Coefficient (log-odds)	Std. Error	t value	p value
Gender (woman)	0.11631112	1.92E-01	6.06E-01	5.45E-01
Residence (rural)	0.06466472	2.46E-01	2.63E-01	7.93E-01
Age (≥35 years)	-0.8369863	2.11E-01	-3.96E+00	7.57E-05
1|2	-1.1297864	2.35E-01	-4.80E+00	1.60E-06
2|3	-0.1166796	2.29E-01	-5.10E-01	6.10E-01
3|4	1.4308197	2.44E-01	5.86E+00	4.54E-09
4|5	2.8669365	3.31E-01	8.66E+00	4.71E-18

Table 24. Predicted probabilities for respondents' concerns about livestock odours in villages (model: Table 23). Only total and statistically significant predictors are shown. Likert-scale categories from 1 "Not concerned at all" *to 5* "Very concerned".

Likert-scale<br>Age	1	2	3	4	5
<35	0.227	0.22	0.344	0.149	0.0589
>=35	0.413	0.246	0.241	0.0735	0.0255
Total	0.3571	0.2384	0.2724	0.0964	0.0356

Table 25. Ordinal logistic regression results on respondents´ opinion that they *feel threatened by shepherds' dogs* on the rural environment, by gender (reference: man), residence (reference: urban) and age (reference: <35 years old) of respondent. Thresholds between Likert-scale categories from 1 *"Completely in disagreement"* to 5 "Completely in agreement*"*.

Predictor / Threshold	Coefficient (log-odds)	Std. Error	t value	p value
Gender (woman)	1.42E-01	1.95E-01	7.30E-01	4.65E-01
Residence (rural)	-1.44E-01	2.59E-01	-5.56E-01	5.78E-01
Age (≥35 years)	-2.29E-01	2.09E-01	-1.10E+00	2.72E-01
1|2	-3.22E-01	2.29E-01	-1.41E+00	1.59E-01
2|3	5.70E-01	2.27E-01	2.50E+00	1.23E-02
3|4	1.75E+00	2.48E-01	7.07E+00	1.54E-12
4|5	3.06E+00	3.26E-01	9.38E+00	6.35E-21

Table 26. Predicted probabilities for respondents´ opinion that they feel threatened by shepherds' dogs on the rural environment (model: Table 25). Only total and statistically significant predictors are shown. Likert-scale categories from 1 *"Completely in disagreement"* to 5 "Completely in agreement*"*.

Likert-scale	1	2	3	4	5
Total	0.448	0.2152	0.2018	0.0941	0.0408

Table 27. Ordinal logistic regression results on respondents´ opinion that herders interfere negatively *on the activity of agricultural farmers*, by gender (reference: man), residence (reference: urban) and age (reference: <35 years old) of respondent. Thresholds between Likert-scale categories from 1 *"Completely in disagreement"* to 5 "Completely in agreement*"*.

Predictor / Threshold	Coefficient (log-odds)	Std. Error	t value	p value
Gender (woman)	2.76E-02	1.97E-01	1.40E-01	8.88E-01

Residence (rural)	2.18E-01	2.59E-01	8.44E-01	3.99E-01
Age (≥35 years)	-5.44E-01	2.10E-01	-2.59E+00	9.63E-03
1|2	-6.00E-01	2.30E-01	-2.61E+00	8.98E-03
2|3	3.39E-01	2.27E-01	1.49E+00	1.35E-01
3|4	2.19E+00	2.74E-01	7.98E+00	1.49E-15
4|5	4.05E+00	4.92E-01	8.24E+00	1.74E-16

Table 28. Predicted probabilities for respondents´ opinion that herders interfere negatively *on the activity of agricultural farmers* (model: Table 27). Only total and statistically significant predictors are shown. Likert-scale categories from 1 *"Completely in disagreement"* to 5 "Completely in agreement*"*.

Likert-scale	1	2	3	4	5
Age					
<35	0.34	0.228	0.325	0.0888	0.0183
>=35	0.475	0.223	0.238	0.0534	0.0105
Total	0.4343	0.2246	0.2642	0.0641	0.0128

Table 29. Ordinal logistic regression results on respondents´ opinion that urban people who visit rural areas do not adapt and try to impose their way of life, by gender (reference: man), residence (reference: urban) and age (reference: <35 years old) of respondent. Thresholds between Likert-scale categories from 1 *"Completely in disagreement"* to 5 "Completely in agreement*"*.

Predictor / Threshold	Coefficient (log-odds)	Std. Error	t value	p value
Gender (woman)	-5.93E-02	1.89E-01	-3.14E-01	7.54E-01
Residence (rural)	4.02E-01	2.49E-01	1.62E+00	1.06E-01
Age (≥35 years)	3.44E-01	2.03E-01	1.70E+00	8.97E-02
1|2	-2.73E+00	3.02E-01	-9.02E+00	1.94E-19
2|3	-1.50E+00	2.37E-01	-6.31E+00	2.86E-10

3|4	-1.88E-01	2.18E-01	-8.64E-01	3.88E-01
4|5	1.26E+00	2.28E-01	5.53E+00	3.28E-08

Table 30. Predicted probabilities for respondents´ opinion that urban people who visit rural areas do not adapt and try to impose their way of life (model: Table 29). Only total and statistically significant predictors are shown. Likert-scale categories from 1 *"Completely in disagreement"* to 5 *"*Completely in agreement*"*.

Likert-scale	1	2	3	4	5
Total	0.0483	0.0989	0.24	0.3385	0.2742
